# Recurrent cross-reactivity between *Candida tropicalis* and *Candida parapsilosis* on the BIOFIRE FilmArray Blood Culture Identification 2 panel

**DOI:** 10.1128/jcm.00335-26

**Published:** 2026-04-30

**Authors:** Emily Puumala, Ryan W. Stevens, Nancy L. Wengenack

**Affiliations:** 1 Division of Clinical Microbiology, Department of Laboratory Medicine and Pathology, Mayo Clinic 6915, Rochester, Minnesota, USA; 2 Department of Pharmacy Services, Mayo Clinic 6915, Rochester, Minnesota, USA; University of Utah, Salt Lake City, Utah, USA

**Keywords:** bloodstream infection, *Candida tropicalis*, *Candida parapsilosis*, multiplex PCR

## LETTER

The BIOFIRE FilmArray Blood Culture Identification 2 (BCID2) panel provides accurate and swift identifications of the most common organisms causing yeast bloodstream infection from positive blood culture bottles; however, performing subculture and reference identification techniques for result confirmation remains imperative (1). We recently published an evaluation of BCID2 performance over a 4-year period and investigated result discrepancies between BCID2 and culture coupled to matrix-assisted laser desorption ionization-time of flight (MALDI-ToF) mass spectrometry (MS) for species identification from blood culture bottles positive for yeast (1). Of 255 yeast identifications, we found three instances where ≥1 yeast was identified by BCID2, followed by single pathogen growth in confirmatory culture (1). In two of these cases, *Candida parapsilosis* and *Candida tropicalis* were simultaneously identified by BCID2, while only *C. parapsilosis* was identified following subculture. Due to the small number of incorrect identifications during our study period, conclusions could not be drawn regarding the significance of these findings; however, since this investigation, an additional case emerged at our hospital demonstrating the same discrepancy. Interestingly, the same patient was previously evaluated at an outside, unaffiliated institution, whose clinical microbiology laboratory observed an identical phenomenon on the same device, prompting us to evaluate the pattern more closely. Furthermore, a recent publication by Imataki and colleagues outlining discordant yeast detections by BCID2 revealed that seven of eight total cases where ≥1 yeast was identified by BCID2 with only one pathogen isolated in confirmatory culture involved *C. parapsilosis* and *C. tropicalis* (2).

**Fig 1:**
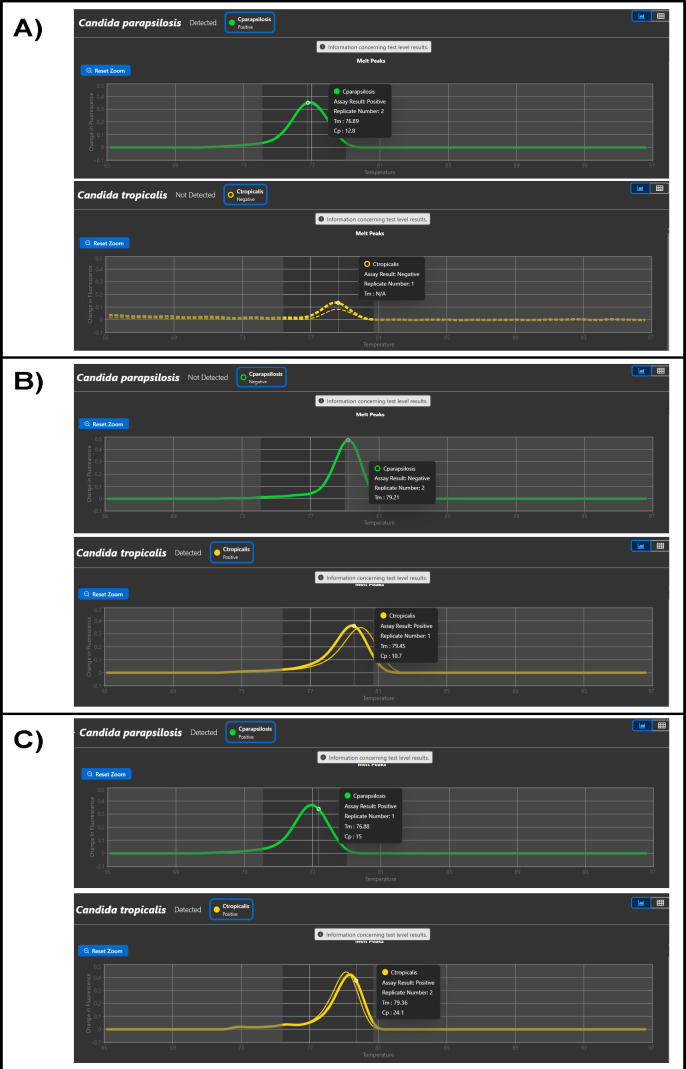
Example melt curve data in the *C. parapsilosis* and *C. tropicalis* assay channels following BCID2 visualized using the BIOFIRE FIREWORKS software for single- and dual-target positive runs. (**A**) *C. parapsilosis* single-target positive BCID2 run (green), which was confirmed by subculture and MALDI-ToF MS. Small peaks are visible in the *C. tropicalis* assay channel (yellow); however, result released was “not detected,” as fluorescent amplification signal was sub-threshold. (**B**) *C. tropicalis* single-target positive BCID2 run (yellow), which was confirmed by subculture and MALDI-ToF MS. Peaks are visible in the *C. parapsilosis* assay channel (green); however, result released was “not detected,” as melting temperatures (Tm) generated following derivation of amplification were outside acceptable parameters. (**C**) Dual-positive run where BCID2 detected both *C. parapsilosis* (green) and *C. tropicalis* (yellow) with acceptable PCR parameters, while subculture and MALDI-ToF MS yielded only *C. parapsilosis*. Amplification curves are not displayed, as the FIREWORKS software does not generate crossing point (Cp) or amplification data when the final interpretation of the assay target is “not detected.”

BioFire Diagnostics (bioMérieux, Inc., Salt Lake City, USA) outlines in the BCID2 package insert, as well as in the corresponding FDA 510(k) Substantial Equivalence Determination Summary, that the assay for *C. parapsilosis* nonspecifically amplifies *C. tropicalis* at high concentrations and vice versa (3, 4). Additionally, a class 2 device recall was issued for BCID2 when used in concert with BD BACTEC (Becton, Dickinson and Company, Sparks, USA) blood culture bottles due to false detections of *C. tropicalis* attributed to nonviable nucleic acids present in blood culture media (5). Although our laboratory does not calculate organism concentrations in positive blood culture bottles, we concluded, based on time to blood culture positivity, discordant co-identifications of *C. parapsilosis* and *C. tropicalis* were likely not correlated to elevated initial inoculum density (6). The average time to positivity for blood cultures yielding a single *C. parapsilosis* BCID2 identification, a single *C. tropicalis* identification, or a co-identification had mean times to positivity of 38.9, 21.2, and 50 h, respectively. Notably, melt curve data generated by the BIOFIRE FilmArray TORCH instrument for each single *C. parapsilosis* (*n* = 20) and *C. tropicalis* (*n* = 5) identification event from our published dataset (1) revealed 90% of *C. parapsilosis*-only identifications demonstrated a melt curve in the *C. tropicalis* channel, while 20% of *C. tropicalis*-only BCID2 results demonstrated a melt curve in the *C. parapsilosis* channel (Fig. 1A and B). This represents a distinction from the earlier class 2 recall, which only references spurious *C. tropicalis* amplifications occurring indiscriminately in positive blood culture bottles, due to the presence of nonviable DNA in culture reagents. Notably, in most cases where *C. parapsilosis* was the only organism detected on BCID2 and confirmatory subculture, an out-of-range peak was observed in the *C. tropicalis* channel (Fig. 1A). These phenomena were not observed in any other yeast or bacterial target channel. As such, it is reasonable to hypothesize that the region of the mitochondrial genome targeted by the primers and probes used by the BCID2 *C. parapsilosis* and *C. tropicalis* PCR assays demonstrates sequence homology not present across the primer-probe bindings sites of the other yeasts targeted (3). Such homology, even if limited, could increase the likelihood of dual positive detections by BCID2 in blood cultures positive for *C. parapsilosis* or *C. tropicalis,* regardless of yeast concentration in culture.

Based on the findings outlined here, we write to recommend that clinical microbiology laboratories using the BCID2 assay interpret *C. parapsilosis* and *C. tropicalis* positive results with caution. Laboratory leadership may consider implementing reporting criteria, especially in cases where *C. parapsilosis* and *C. tropicalis* are identified in tandem, where a general “*Candida* species” result is reported, while confirmatory subculture and mass spectrometry or sequencing are performed for species-level identification.
